# Severe Fever with Thrombocytopenia Syndrome Virus Antigen Detection Using Monoclonal Antibodies to the Nucleocapsid Protein

**DOI:** 10.1371/journal.pntd.0004595

**Published:** 2016-04-05

**Authors:** Aiko Fukuma, Shuetsu Fukushi, Tomoki Yoshikawa, Hideki Tani, Satoshi Taniguchi, Takeshi Kurosu, Kazutaka Egawa, Yuto Suda, Harpal Singh, Taro Nomachi, Mutsuyo Gokuden, Katsuyuki Ando, Kouji Kida, Miki Kan, Nobuyuki Kato, Akira Yoshikawa, Hiroaki Kitamoto, Yuko Sato, Tadaki Suzuki, Hideki Hasegawa, Shigeru Morikawa, Masayuki Shimojima, Masayuki Saijo

**Affiliations:** 1 Department of Virology I, National Institute of Infectious Diseases, Tokyo, Japan; 2 Miyazaki Prefectural Institute for Public Health and Environment, Miyazaki, Japan; 3 Kagoshima Prefectural Institute for Environmental Research and Public Health, Kagoshima, Japan; 4 Saga Prefectural Institute of Public Health and Pharmaceutical Research, Saga, Japan; 5 Okayama Prefectural Institute for Public Health and Environmental Science, Okayama, Japan; 6 Ehime Prefectural Institute of Public Health and Environmental Science, Ehime, Japan; 7 Health and Sanitation Office, Tottori Prefecture Institute of Public Health and Environmental Science, Tottori, Japan; 8 Nagasaki Prefectural Institute for Environmental Research and Public Health, Public Health Science Research Center, Nagasaki, Japan; 9 Hyogo Prefectural Institute of Public Health and Consumer Sciences, Hyogo-ku Kobe-shi, Japan; 10 Department of Pathology, National Institute of Infectious Diseases, Tokyo, Japan; 11 Department of Veterinary Science, National Institute of Infectious Diseases, Tokyo, Japan; Florida Gulf Coast University, UNITED STATES

## Abstract

**Background:**

Severe fever with thrombocytopenia syndrome (SFTS) is a tick-borne infectious disease with a high case fatality rate, and is caused by the SFTS virus (SFTSV). SFTS is endemic to China, South Korea, and Japan. The viral RNA level in sera of patients with SFTS is known to be strongly associated with outcomes. Virological SFTS diagnosis with high sensitivity and specificity are required in disease endemic areas.

**Methodology/Principal Findings:**

We generated novel monoclonal antibodies (MAbs) against the SFTSV nucleocapsid (N) protein and developed a sandwich antigen (Ag)-capture enzyme-linked immunosorbent assay (ELISA) for the detection of N protein of SFTSV using MAb and polyclonal antibody as capture and detection antibodies, respectively. The Ag-capture system was capable of detecting at least 350–1220 TCID_50_/100 μl/well from the culture supernatants of various SFTSV strains. The efficacy of the Ag-capture ELISA in SFTS diagnosis was evaluated using serum samples collected from patients suspected of having SFTS in Japan. All 24 serum samples (100%) containing high copy numbers of viral RNA (>10^5^ copies/ml) showed a positive reaction in the Ag-capture ELISA, whereas 12 out of 15 serum samples (80%) containing low copy numbers of viral RNA (<10^5^ copies/ml) showed a negative reaction in the Ag-capture ELISA. Among these Ag-capture ELISA-negative 12 samples, 9 (75%) were positive for IgG antibodies against SFTSV.

**Conclusions:**

The newly developed Ag-capture ELISA is useful for SFTS diagnosis in acute phase patients with high levels of viremia.

## Author Summary

Severe fever with thrombocytopenia syndrome (SFTS) is a tick-borne emerging infectious disease caused by a novel bunyavirus, SFTS virus (SFTSV). Since first discovered in China in 2011, SFTSV has been detected from SFTS patients and ticks with expanding geographic ranges from China to Japan and South Korea. The potential for SFTS spread to other warm or sub-tropical regions makes it a serious concern to the public health. It is of great importance to detect SFTSV rapidly and specifically for the effective control of the disease. For the diagnosis of viral infections, a sandwich antigen (Ag)-capture ELISA detecting viral nucleoprotein (N) in viremic serum samples has been widely applied to detect the agents, since it is the most abundant viral antigen and has highly conserved amino acid sequence. In this study, using the novel monoclonal antibodies raised against SFSTV-N, an Ag-capture ELISA system was developed, and the validation of this system was performed using sera collected from SFTS-suspected patients. Our data show that the Ag-capture ELISA was useful for the diagnosis of SFTS patients in the acute phase of the disease. This study shows a novel methodology for the diagnosis of SFTS, which may provide helpful information for the effective control of the disease.

## Introduction

Between 2007 and 2010, a severe febrile illness associated with gastrointestinal symptoms, thrombocytopenia, and leukocytopenia caused by an unknown etiological agent was reported in rural areas of Hubei and Henan provinces in Central China [1]. The case-fatality rate of the disease was reported to be between 12%–30% at that time. The disease was named severe fever with thrombocytopenia syndrome (SFTS), or fever, thrombocytopenia and leukopenia syndrome (FTLS) [1,2]. A novel phlebovirus, termed SFTS virus (SFTSV and also known as Huaiyangshan virus or Henan Fever Virus), has been identified as the causative agent of the disease [1,2,3]. SFTSV has been detected in two tick species (*Haemaphysalis longicornis* and *Rhipicephalus microplus*) in epidemic areas, suggesting that these ticks are the most likely vectors for transmission of the virus to humans [1,3]. SFTSV antibodies were detected at various rates in goats, cattle, sheep, pigs, dogs, and chickens [4,5,6,7,8,9], indicating that these animals were infected with SFTSV. There are no reports describing that the virus causes disease in these animals, suggesting that these animals and some species of ticks are the reservoirs of SFTSV. SFTS is endemic to Japan and South Korea [10,11]. SFTS patients show abrupt onset of fever with gastrointestinal tract symptoms in the early phase. Most patients have marked thrombocytopenia and leukocytopenia at this stage. Later stages of the syndrome are characterized by a progressive multiple organ failure in fatal cases or a self-limiting process in survivors [12]. The level of viral RNA in patient sera correlates to the clinical outcome. In fatal cases, viremia increases to 10^9^ viral copies per mL. In contrast, the convalescent stage is characterized by decreasing levels of viremia and normalization of clinical laboratory parameters [13,14,15].

SFTSV is classified into the genus *Phlebovirus*, family *Bunyaviridae*. Tick-borne phleboviruses (TBPVs) including SFTSV are globally distributed. TBPVs closely related to SFTSV, such as Heartland virus, Malsoor virus, and Hunter Island Group viruses, have been discovered [16,17,18]. Phylogenetic and serological studies revealed that TBPVs are classified into four distinct groups, Uukuniemi group, Bhanja group, SFTS/Heartland virus group, and Kaisodi group [19,20]. SFTSV is classified into the SFTS/Heartland virus group.

SFTSV has 3 segmented, single-stranded RNA genomes and is composed of large (L), medium (M), and small (S) segments. The L, M, and S segments encode an RNA-dependent RNA polymerase, a precursor of glycoproteins (Gn and Gc), a nucleocapsid (N) protein and a nonstructural (NS) protein using an ambisense coding strategy, respectively [1]. The N protein is highly immunogenic and conserved among all isolates in each of the phleboviruses [21,22]. Therefore, N protein is often selected as a target of antigen (Ag) and antibody detection [23,24,25].

SFTS and other infectious diseases are difficult to diagnose clinically without microbiological tests, particularly when symptoms are non-specific. Hence, laboratory tests are necessary for SFTS diagnosis. Several genome amplification-based methods for SFTS diagnosis have been reported e.g., conventional reverse transcription-PCR (RT-PCR), quantitative RT-PCR, reverse transcription-loop-mediated isothermal amplification assay (RT-LAMP), and reverse transcription-cross-priming amplification coupled with vertical flow visualization [2,13,26,27,28]. However, genome amplification techniques are limited by their requirement of expensive equipment and technical expertise. Methods for the detection of viral Ags using an Ag-capture sandwich ELISA have been previously described, and the sensitivity of this assay is comparable to that of RT-PCR for the detection of Lassa virus and filoviruses [23,24,29,30,31,32,33]. The assay is highly accurate in identifying viral Ags in a rapid and robust manner; additionally, it has been accepted as a useful method for diagnosis during the acute phase of viral infections. To our knowledge, an Ag-capture sandwich ELISA has not yet been developed for SFTS.

In this study, mouse MAbs against SFTSV N protein were generated and characterized. Furthermore, Ag-capture ELISA for detection of SFTSV using the MAb was developed, and its efficacy in SFTS diagnosis was evaluated using sera collected from patients with SFTS.

## Materials and Methods

### Ethics statement

All samples were collected as part of public health diagnostic activities for SFTS, were pre-existing relative to the start of the study, and were examined as anonymous samples. All protocols and procedures were approved by the research ethics committee of the National Institute of Infectious Diseases for the use of human subjects (no. 531).

The experiments with animals were performed in strict accordance with the Animal Experimentation Guidelines of the National Institute of Infectious Diseases. The protocol of experiments for mice and rabbits were approved by the Institutional Animal Care and Use Committee of the National Institute of Infectious Diseases (Permit number: 112116 and 111124, respectively).

### Cell culture

Mouse myeloma cells, P3X63Ag8.653, obtained from the American Type Culture Collection (Manassas, VA), were maintained in RPMI 1640 medium (Sigma-Aldrich, St. Louis, MO) supplemented with 10% heat-inactivated fetal bovine serum (FBS) and kanamycin sulfate (Life Technologies, Carlsbad, CA). Hybridomas were maintained in Growth Medium E (Stem Cell Technologies, Vancouver, Canada), RPMI 1640 medium supplemented with 10% FBS and kanamycin sulfate, or KBM-343 medium (Kohjin Bio Co., Ltd., Saitama, Japan) supplemented with antibiotics. BTI-TN-5B1-4 (Tn5, High Five; Life Technologies) insect cells were maintained in TC100 (Life Technologies) supplemented with 10% FBS, 2% tryptose phosphate broth (Difco, Detroit, MI), and kanamycin sulfate. Huh7, Vero, and Vero E6 cells, obtained from the American Type Culture Collection, were maintained in DMEM (Sigma-Aldrich) supplemented with 5% FBS and kanamycin sulfate.

### Viruses

SFTSV strains YG1, SPL004, and SPL010 isolated from serum samples of Japanese patients with SFTS were used [11]. SFTSV strain HB29 was kindly provided by De-Xin Li and MiFang Liang, Chinese Center for Disease Control and Prevention, Beijing, People’s Republic of China. As a negative control antigen, a supernatant of Vero E6 cells infected with Rift Valley fever virus (RVFV) strain MP-12 was used [23]. Experiments using infectious SFTSV and RVFV were conducted in a biosafety level (BSL)-3 laboratory. Forecariah virus (FORV) and Palma virus (PALV), which were kindly gifted from Robert Tesh, University of Texas Medical Branch, USA, were handled in BSL-2. The infectious dose of the SFTSV and RVFV stock solutions was determined by calculating the 50% tissue culture infectious dose (TCID_50_) as described previously [11,13,34]. Preliminary experiments indicated that treatment of sera containing SFTSV (10^7^ TCID_50_/ml) with 1% triton X-100 in combination with UV-irradiation (312 nm, 2.5 mW/cm^2^) on a trans-illuminator for 10 min caused complete loss of viral infectivity in cells. Therefore, viruses used for Ag-capture ELISA were treated with UV-irradiation on a trans-illuminator for 10 min and followed by 1% Triton X-100 for the destruction of the virus particle.

### Serum samples

We asked medical personnel in Japan to inform us on a voluntary basis if they had seen any patients with symptoms similar to those of SFTS, as reported previously [11]. Through the courtesy of prefectural and municipal public health institutes, 63 serum samples were collected from 55 acute phase patients suspected of SFTS in Japan. Viral gene detection by the qRT-PCR and viral antibody detection by IgG ELISA and/or IFA were conducted to diagnose SFTS. From 55 patients, 34 of these were diagnosed as having SFTSV. Serum samples obtained from 18 healthy donors were used to establish the cut-off value of the IgG ELISA. Serum samples used for IgG ELISA were inactivated under the UV light in the biosafety cabinet for 1 h. Serum samples used for Ag-capture ELISA were treated with 1% Triton X-100 for the destruction of the virus particle followed by an UV-irradiation on a trans-illuminator for 10 min.

### Recombinant baculoviruses

The recombinant baculovirus, Ac-His-SFTSV-N expressing a histidine (His)-tagged SFTSV recombinant N (rN) protein at C-terminal, was generated as described previously [23,35,36]. Briefly, the cDNA encoding the N protein of SFTSV strain HB29 (nucleotide position 43–780 of segment S, GenBank accession No. NC_018137) was artificially synthesized (GeneScript, Piscataway, NJ) and then was ligated into the BamHI sites upstream of the 8-His tag coding sequence of the transfer vector pAcYM1 [37]. Tn5 cells were transfected with mixtures of the transfer vector pAcYM1-SFTSV-N and BD BaculoGold Linearized Baculovirus DNA (BD Biosciences, San Jose, CA) according to the manufacturer’s instructions with the procedures described by Kitts et al. [38] but with modification by Matsuura et al. [37]. A baculovirus (Ac-ΔP), which lacks polyhedrin expression, was used as a negative control virus.

### Expression and purification of recombinant N proteins

SFTSV rN protein [>75% purity as determined by ImageJ analysis (http://rsbweb.nih.gov/ij/) of sodium dodecyl sulfate polyacrylamide gel electrophoresis] was generated as previously described [23,35,36]. Briefly, Tn5 cells infected with Ac-His-SFTSV-N were incubated at 27°C for 72 h. The cells were then washed three times with phosphate-buffered saline (PBS) solution. The cells were lysed in PBS solution containing 1% Nonidet P-40 (NP-40) and sonicated. After the cell lysates were centrifuged at 17,800 × g for 10 min at 4°C, the supernatant fractions were collected as a source of SFTSV rN protein for purification. SFTSV rN protein was purified by Ni^2+^-nitrilotriacetic acid affinity chromatography (Qiagen, Hilden, Germany) according to the manufacturer’s instructions. RVFV rN protein was expressed and purified as described previously [23]. The histidine-tag was not removed from the rN protein. The concentration of the purified SFTSV and RVFV rN proteins were determined by the Pierce BCA Protein Assay Reagent (Life Technologies). Antigens were aliquoted and stored at −80°C until use.

### Production of MAbs

BALB/c mice were immunized subcutaneously twice with the purified SFTSV rN protein emulsified in TiterMAX Gold (TiterMax USA, Inc., Norcross, GA, USA). Hybridomas were produced by fusion of myeloma cells with the splenic cells, obtained 4 days after the last immunization, using ClonaCell-HY Hybridoma Kit (Stem Cell Technologies) according to the manufacturer’s instructions. The culture supernatants of hybridoma cells were screened for the presence of antibodies against SFTSV antigen by IgG ELISA as described below. Positive hybridoma cells were cloned by limiting dilution. The isotypes of the MAbs were determined using Mouse Monoclonal Antibody Isotyping Kit (AbD Serotec, Kidlington, UK). The MAbs were purified from mouse ascitic fluid (Unitech Co. Ltd., Chiba, Japan) or from the culture supernatant by protein G column chromatography (MAbTrap Kit, GE Healthcare UK Ltd., Buckinghamshire, UK) according to the manufacturer’s instructions. The concentration of each purified MAb was determined using the Pierce BCA Protein Assay Reagent. Two hybridoma clones (designated as 2D11 and 9D3) producing MAbs reactive to SFTSV N protein were obtained. MAb 9D3 and MAb 2D11 were isotypes of IgG_1_ and IgG_2a_, respectively. The light chain of these MAbs was κ-type.

### Rabbit polyclonal antibody to each of the SFTSV and RVFV rN proteins

Polyclonal antibodies to each of the rN proteins of SFTSV and RVFV were raised by immunization of rabbits with the respective rN protein [11,23]. Polyclonal antibodies to FORV and PALV were produced by infection of rabbits with FORV and PALV, respectively. Rabbit sera were obtained 7 days after infection. The experiments with animals were performed in strict accordance with the Animal Experimentation Guidelines of the National Institute of Infectious Diseases.

### Immunofluorescence assay (IFA)

The antigens of SFTSV strain YG1, FORV, PALV, and RVFV were prepared for IFA as previously described [39]. Briefly, Vero cells infected with each virus (MOI = 0.1) were cultured, harvested by trypsinization, washed with PBS, and mixed with parent uninfected cells at a ratio of 1:3. The cells were spotted on to 14-well HT-coated slide glasses (AR Brown Co., Ltd., Tokyo, Japan), air dried, and fixed with a mixture of methanol and acetone [1:1 (v/v)]. These IFA antigen slides were stored at -80°C until use. They were thawed and dried immediately prior to use. The IFA was performed by diluting MAbs at the concentration of 1 ng/μl with PBS and were placed on the slides. As a positive control, rabbit sera diluted with PBS at 1:1,000 were also placed on the slides. The slides were incubated under humidified conditions at 37°C for 1 h. After washing with PBS, the slides were treated with Alexa Fluor 488 conjugated goat anti-mouse IgG (H + L) antibody (Life Technologies) or Alexa Fluor 488 conjugated goat anti-rabbit IgG (H + L) antibody (Life Technologies) diluted with PBS at 1:400. The slides were incubated under humidified conditions at 37°C for 1 h. After washing, the slides were examined for antigen staining under a fluorescent microscope (Olympus, Tokyo, Japan) [11,39].

### Immunohistochemistry (IHC)

Immunohistochemical analysis was performed as previously described [11]. The mouse MAbs 9D3 and 2D11 were used in the immunohistochemical analysis as the primary antibodies. Lymph nodes of necrotizing lymphadenitis without SFTSV infection were used as negative controls for tissue specimens.

### IgG ELISA

The IgG ELISA was performed as previously described, except for antigen preparation [32,33,35,36]. Antigen preparation for IgG ELISA was performed by infecting Huh7 cells with SFTSV strain HB29 (MOI = 0.1) and incubated at 37°C for 48 h. The cells were collected and washed with PBS, and then lysed with PBS solution containing 1% NP-40. The cell lysates were centrifuged at 8,000 rpm for 10 min at 4°C, and the supernatant fraction was collected as a source of SFTSV antigen for IgG ELISA. Huh7 cell lysates without infection were treated in the same way as that for SFTSV antigen preparation and were used as a negative control antigen. Nunc-Immuno Plates (Thermo Fisher Scientific Inc., Waltham, MA) were coated with a pre-determined optimal quantity of Huh7 cell lysates prepared from SFTSV-infected or uninfected cells diluted with PBS at 1:800 and incubated at 4°C overnight. The following procedure was performed in the same way as described previously [32]. The cut-off value was set as the average value of the control sera (healthy donor sera) plus three times standard deviation (SD; mean + 3×SD). The sample was considered positive if it yielded an OD_405_ value above the cut-off value.

### Ag-capture ELISA

The Ag-capture ELISA was performed as previously described [23,24,29]. Nunc-Immuno plates were coated with 100 ng of capture MAbs (9D3 or 2D11) in 100 μl of PBS at 4°C overnight, and then the wells were incubated with a blocking reagent. After removal of the blocking solution, a series of samples (100 μl/well) diluted with PBST-M was added and incubated for 2 h at RT. After the plates were washed, 100 μl of the rabbit anti-SFTSV rN protein sera diluted 1:1,000 with PBST-M was added to each well, followed by incubation for 2 h at RT. After washing, HRP-conjugated goat anti-rabbit IgG antibody (Life Technologies) diluted 1:1,000 with PBST-M were added to each well and incubated for 2 h at RT. After further washing, 100 μl of ABTS [2,2azinobis (3-ethylbenzthiazolinesulfonic acid)] substrate solution (Roche Applied Science, Penzberg, Germany) was added and incubated for 30 min at RT. The optical density at 405 nm (OD_405_) was measured against a reference of 490 nm using a microplate reader (Model 680 Microplate Reader; Bio-Rad Laboratories Inc., Hercules, CA). The adjusted OD_405_ value was calculated by subtracting the OD_405_ value of the negative antigen-coated wells from that of the corresponding wells. The cut-off value was set at the average value of the control sera (antigen free) plus three times the standard deviation (SD; mean + 3×SD). The sample, which yielded an OD_405_ value above the cut-off value, was thus considered positive. Protein or viral quantities detected per 100 μL reaction in 96-well micro-plates in the Ag-capture ELISA were presented as “/100 μL/well.”

### Quantitative one-step RT-PCR (qRT-PCR)

The qRT-PCR method using the qRT-PCR primer and probe sets targeted to N protein or glycoprotein genes was performed as described previously [13]. Genome copies obtained from qPCR assays were presented as “/mL” of serum samples.

### Statistical analysis

Unpaired *t*-test with Welch's correction was used to determine significant differences in the data using the GraphPad Prism 6 software program (GraphPad software, La Jolla, CA). A significant difference was considered to be present for any p value <0.05.

## Results

### Reactvity of MAbs to SFTSV N protein

**Fig 1:**
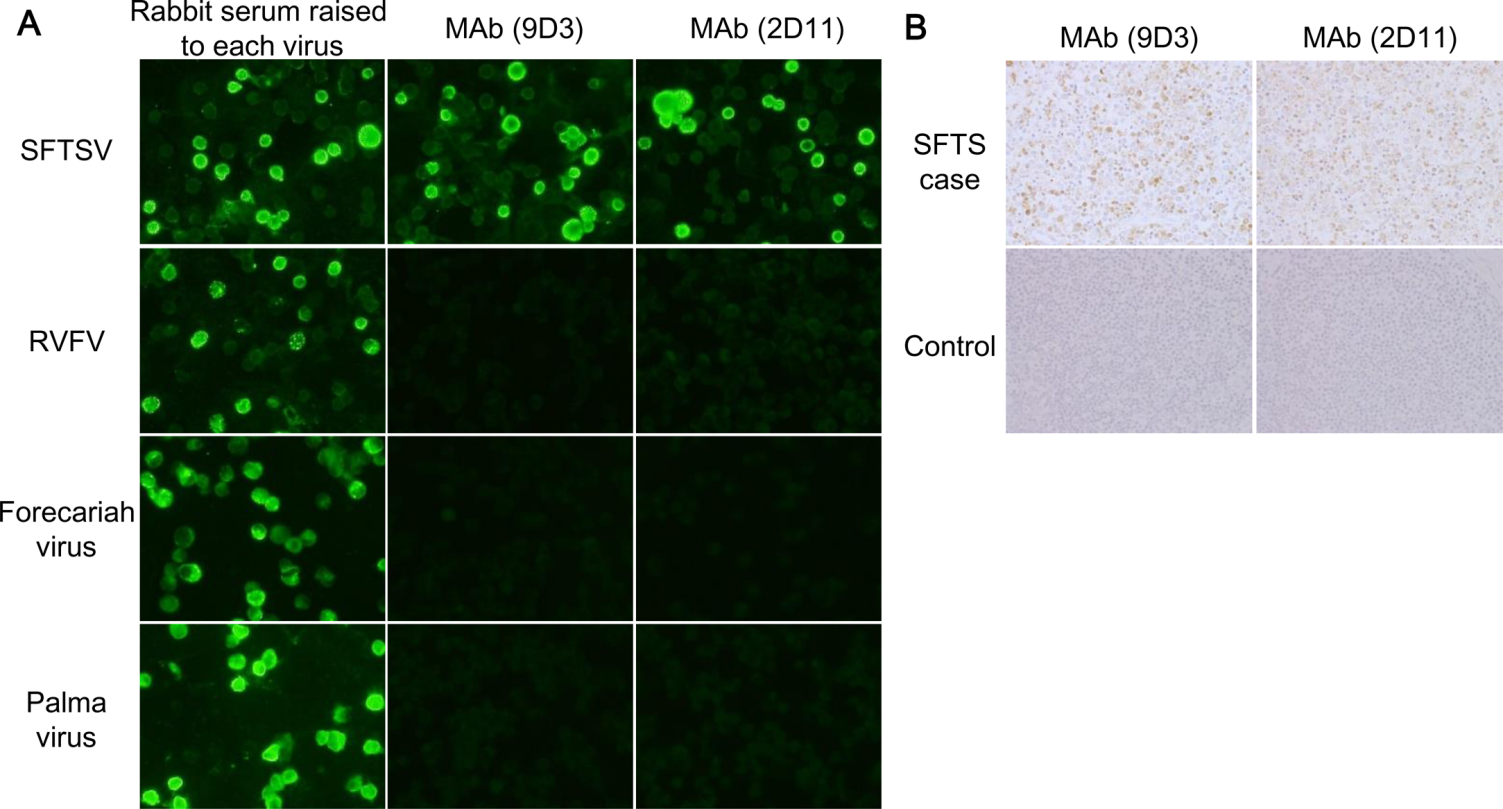
Reactivity of MAbs (9D3 and 2D11) to SFTSV N protein and other TBPVs. (A) The indirect immunofluorescence staining (IFA) of MAbs. Vero cells infected with SFTSV strain YG1, RVFV strain MP12, FORV, and PALV were stained with MAbs. Rabbit sera obtained from animals immunized with SFTSV or RVFV recombinant N protein, or infected with FORV or PALV, were used as positive controls in the IFA. (B) The immunohistochemical staining of SFTSV antigens with the developed MAbs. The lymph nodes collected from patient with SFTS and patients without SFTS were used for evaluation of utility of the MAbs in SFTS diagnosis with the IHC analysis.

Novel MAbs (9D3 and 2D11) against SFTSV N were generated in this study. SFTSV N protein characterized by a diffuse granular cytoplasmic staining was detected by these MAbs through indirect immunofluorescence (IFA) for SFTSV infected Vero cells, but was not detected in Rift Valley fever virus (RVFV) infected cells (Fig 1A). Since the serologic relationships between SFTSV and Bhanja group virus, including Forecariah virus (FORV) and Palma virus (PALV), have been demonstrated [19], we also examined the cross-reactivity of MAbs to these phleboviruses. As shown Fig 1A, both MAbs did not react to FORV and PALV. SFTSV antigens were detected in the lymph node specimens obtained from patients with SFTS clearly through immunohistochemistry (IHC) staining using the MAbs, but not in that of the patients without SFTS (Fig 1B).

### Detection of SFTSV rN protein and authentic SFTSV by the developed Ag-capture ELISA

**Fig 2:**
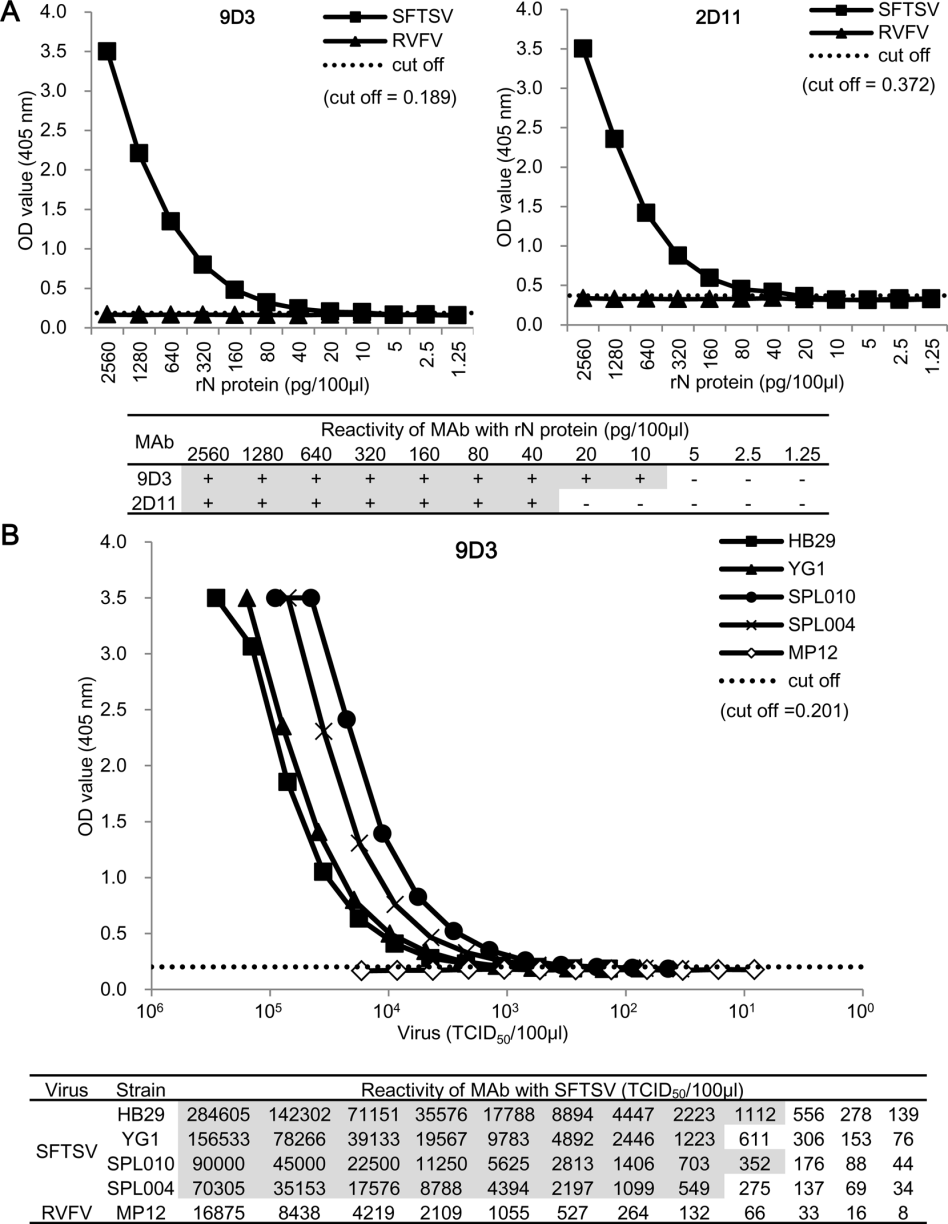
Detection limit and cross reactivity of the Ag-capture ELISA. (A) The detection limit of SFTSV rN protein by the Ag-capture ELISA using MAb 9D3 or 2D11. (B) The detection of authentic SFTSV by the Ag-capture ELISA. The Ag-capture ELISA were used MAb 9D3 or 2D11 as capture antibodies and anti-rN protein rabbit serum as detecting antibody. The dashed lines indicate the cut-off values (mean + 3×SD derived from OD_405_ values without antigens) for each ELISA. The detection limits for each MAb are shown in the lower panel.

The minimum amounts of SFTSV rN protein detected in the Ag-capture ELISA with MAb 2D11 and MAb 9D3 were 40 pg and 10 pg /100 μL/well, respectively (Fig 2A), while levels of up to 2.6 ng of RVFV rN protein were not detected (Fig 2A). The Ag-capture ELISA using MAb 9D3 was more sensitive in detection of SFTSV rN protein than the MAb 2D11. The Ag-capture ELISA using both MAbs (9D3 and 2D11) as capture antibody was less sensitive than using MAb 9D3 alone (S1 Fig). Therefore, the MAb 9D3-based Ag-capture ELISA was selected for further experiments. Four SFTSV strains, including a Chinese strain (HB29) that we tested were detected in the Ag-capture ELISA. The minimum levels of SFTSV detected in the assay were 1,100, 1,200, 350, and 540 TCID_50_ /100 μL/well for SFTSV strains HB29, YG1, SPL010, and SPL004, respectively (Fig 2B). Because 1.0 TCID_50_ of SFTSV corresponds to approximately 15.4 copies of the SFTSV genome [13], the sera containing theoretical value of more than 5,000 copies of the SFTSV genome/100 μL/well could be used for Ag detection in this assay. In contrast, RVFV antigens prepared from virus infected culture supernatants were not detected in this assay.

### Efficacy of the Ag-capture ELISA in SFTS diagnosis

**Fig 3:**
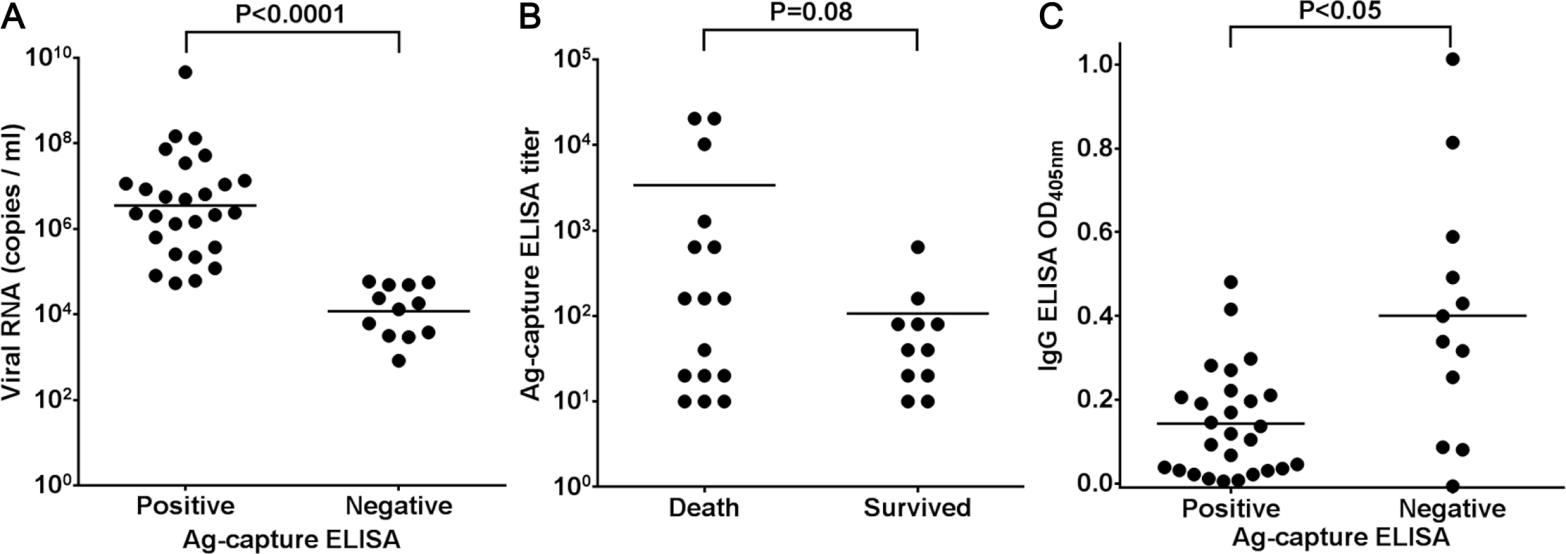
Correlation between the results of the Ag-capture ELISA and that of the viral RNA copy numbers or OD values of IgG ELISA. (A) Correlation between the result of the Ag-capture ELISA and viral RNA copy numbers in the serum samples. The Ag-capture ELISA (x axis) and the viral RNA copy number determined by qRT-PCR (y axis) from each sample are plotted as dots. (B) Relationship between the titers of the Ag-capture ELISA and the patients' outcomes. The patients' outcomes (x axis) and the titers determined by the Ag-capture ELISA (y axis) from each sample are plotted as dots. (C) Correlation between the result of the Ag-capture ELISA and OD values of IgG ELISA. The results determined by the Ag-capture ELISA (x axis) and OD_405_ values determined by IgG ELISA (y axis) from each sample are plotted as dots. The mean of each group is indicated by a horizontal bar. The *t*-test was used to determine the level of statistical significance. The calculated p values are shown above the groups that were compared.

In order to evaluate the efficacy of the Ag-capture ELISA in SFTS diagnosis, these systems were tested using acute phase sera collected from patients suspected of having SFTS. In a total of 63 serum samples, 24 samples were negative by qRT-PCR, and they were also negative for IgG antibodies to SFTSV determined by IgG ELISA and IFA. The patients, whose sera were negative for virus genome by qRT-PCR and IgG antibodies to SFTSV, were confirmed to be patients without SFTS. In a total of 63 serum samples, 27 samples showed a positive reaction in the Ag-capture ELISA (Table 1). Thirty-nine samples including all the Ag-capture ELISA-positive samples were SFTSV genome positive in the qRT-PCR. All 24 samples containing SFTSV genome copy numbers higher than 10^5^ copies/ml showed a positive reaction in the Ag-capture ELISA, while only 3 of 15 SFTS-genome positive samples with the viral RNA copy numbers of less the 10^5^ copies/ml had a positive reaction in the assay. The sensitivity and the specificity of the Ag-capture ELISA were 69% (27/39) and 100% (24/24), respectively, based on the qRT-PCR results. The viral RNA copy number in the Ag-capture ELISA-positive samples (mean ± SD: 6.548 ± 0.227 log_10_ copies/ml) was significantly higher than that observed in the Ag-capture ELISA-negative samples (4.077 ± 0.178 log_10_ copies/ml; p < 0.0001; Fig 3A).

**Table 1:** The relationship of the results with the Ag-capture ELISA and qRT-PCR tests using SFTS-suspected patient serum samples.

	qRT-PCR			
	Positive(+)		Negative (−)	
	Viral RNA (copies/ml)			
Ag capture ELISA	≥10^5^	<10^5^		Total
Positive (+)	24	3	0	27
Negative (−)	0	12	24	36
Total	24	15	24	63

We then determined the antigen titers of each of the serum samples by using serially-diluted serum samples for the Ag-capture ELISA. There was no statistically significant difference in the antigen titers between patients with SFTS with fatal and non-fatal outcomes (p = 0.08; Fig 3B). However, high antigen titers (≥160) were detected in 82% (9/11) of serum samples collected from patients with fatal outcomes, and only 18% (2/11) of serum samples collected from patients with non-fatal outcomes were detected (Table 2). Furthermore, significant high antigen titers (≥10,240) were detected in serum samples collected from three patients with fatal outcomes (Table 2).

**Table 2:** Results of the Ag-capture ELISA in the qRT-PCR-positive serum samples.

Patient ID^a^	Outcome	Collection time (days after onset)^b^	viral RNA^c^ (log_10_ copies/ml)	Ag-capture ELISA titer	IgG ELISA^d^
003	Death	unknown	5.41	160	+
004	Death	unknown	7.13	640	+
005	Death	unknown	6.75	>20,480	+
010	Death	5	6.30	1,280	-
032	Survived	5	4.73	80	-
053	Death	unknown	7.72	>20,480	-
054	Death	5	4.38	-	-
		8	3.79*	-	+
		9	3.50*	-	+
060	Survived	4	4.91	20	-
062	Survived	4	5.80	80	+
070	Death	5	6.17	20	-
075	Death	2	6.36	10	-
078	Death	8	5.34	10	+
		9	4.69	-	-
097	Survived	6	5.08	40	+
		9	2.92	-	+
105	Survived	3	5.57	80	-
106	Survived	3	6.12	10	-
107	Survived	4	6.33	10	+
108	Death	3	6.93	20	+
114	Survived	8	3.58	-	+
117	Death	3	8.17	10	+
120	Death	0	4.75	-	+
		7	9.67	10,240	-
123	Survived	7	3.47	-	-
124	Death	7	6.38	40	+
127	Survived	4	4.26	-	+
129	Survived	0	6.81	20	-
130	Death	8	7.54	640	+
132	Death	0	7.06	20	-
134	Survived	2	4.79	40	+
137	Survived	7	6.69	160	-
141	Death	0	8.12	160	+
142	Death	5	7.87	160	+
143	Survived	4	4.77	-	-
145	Survived	6	4.12	-	-
146	Survived	6	7.04	640	-
147	Survived	9	4.69	-	-

a ID, identification.

b The time (days) of blood sampling after the onset of illness

c The log_10_ viral RNA copy number of nucleocapsid protein (*glycoprotein)

d +: detected, -: not detected.

We performed the IgG ELISA against SFTSV in the samples to determine the antibody responses, because the presence of the antibodies against SFTSV might inhibit the capture capacity of the MAb for SFTSV N protein. The IgG antibody status was compared between the Ag-capture ELISA positive and negative groups among the total of 39 serum samples positive for qRT-PCR. In 27 Ag-capture ELISA-positive samples, 11 (41%) samples were IgG ELISA-positive, while 9 of 12 (75%) Ag-capture ELISA-negative samples were IgG ELISA-positive. The OD values of the Ag-capture ELISA-positive samples in IgG ELISA (mean ± SD; 0.143 ± 0.024) were significantly lower than those of Ag-capture ELISA-negative samples in the assay (mean ± SD; 0.401 ± 0.087; p < 0.05).

## Discussion

We demonstrated that both the novel 2 MAbs (9D3 and 2D11) generated in this study reacted to SFTSV, but not to RVFV, FORV, and PALV in the genus Phlebovirus (Fig 1A). However, a close antigenic relationship between FORV, PALV and SFTSV was demonstrated by the serological tests [19]. In addition, these MAbs did not react to the recombinant N protein of Heartland virus in IFA (S2 Fig). Therefore, we speculate that the MAbs may not be cross-reactive to Malsoor virus and Hunter Island Group virus, which are closely related to SFTSV. This is because the amino acid sequence homology of N protein of SFTSV strain HB29 with those of Hearland virus, Malosoor virus, and Hunter Island Group virus were shown to be 61.6%, 55.6%, and 60.9%, respectively [20]. As amino acid sequence identities among the N protein of SFTSV strains available from databases are conserved with more than 98% homology, it is thought that the N protein of the Japanese strains and also the Chinese strains and South Korea strains are detectable in the Ag-capture ELISA and IHC using the MAbs developed in the present study. Furthermore, MAbs may be useful for future development of rapid dipstick, flow-through devices that require minimal training and do not require electricity.

The rN protein concentration detectable using the Ag-capture ELISA for detecting SFTSV (10–40 pg /100 μL well) was the same level as that for detecting RVFV with the previously developed Ag-capture ELISA [23]. However, the detection limit of the concentration of authentic viral antigens detectable by the Ag-capture ELISA for SFTSV (350–1,200 TCID_50_/100 μL/well seems to be higher than that for RVFV (7.8–31.3 pfu /100 μL/well) [23]. Although it is difficult to simply compare the detection limits between the two ELISAs, a more sensitive detection of RVFV in Ag-capture ELISA in the previous study may be due to an abundant non-virion associated N protein in the viral supernatants collected from infected cell cultures that exhibit an obvious CPE as described by Shafagati et al [40]. In contrast, since SFTSV do not exhibit obvious CPE on infected Vero cells. Therefore, relatively lower detection limits of authentic SFTSV N protein in the Ag-capture ELISA seems to be attributable to a low amount of non-virion associated SFTSV N protein in the viral supernatants, despite virions being lysed by treatment with the detergent, Triton X-100.

The viral RNA level in sera of patients with SFTS was reported to be associated with the outcomes [13,14,15]. During the first stage of the disease (day 1 to 7 post-onset of illness, taking the day on which symptoms, fever, first appeared as day 0), the serum viral load is high (average 10^5^–10^6^ copies/ml) regardless of the outcomes of fatal or non-fatal cases [41]. During the second stage of the disease (day 7 to 13 post-onset of illness), the serum viral load decreased in non-fatal cases but still remained high in fatal cases (average 10^8^ copies/ml) [41]. It has also been reported that the amounts of Ag detected by the Ag-capture ELISA are well correlated with viremia of ebolavirus or Lassa virus following experimental animal infection [32,42]. Also, a moderate difference has been demonstrated in the serum ebolavirus Ag levels between patients who died and those who survived [43]. These findings suggested that the patient outcomes were expected from the results of Ag-capture ELISA. Indeed, we found that high antigen titers (≥160) were detected at a higher rate in serum samples collected from patients with fatal outcomes than from serum samples collected from patients with non-fatal outcomes (Table 2). However, there was no statistically significant difference in antigen titers between patients with SFTS with fatal and non-fatal outcomes (p = 0.08; Fig 3B). This might be due to the small-scale samples used in this study. Thus, further large-scale investigation is required to elucidate the correlation between the results of Ag-capture ELISA and patient outcomes.

Among qRT-PCR positive-patients, the Ag-capture ELISA-negative patients showed significantly higher IgG responses than the Ag-capture ELISA-positive patients (Fig 3C). We speculate that the amount of N protein in serum samples collected may be lower than the detectin limit of the Ag-capture ELISA, since these patients had already reached convalescence phase, where IgG antibodies to SFTSV N protein had been induced. The induced antibodies against SFTSV N protein in serum samples may inhibit the reaction of the MAb the N protein in the Ag-capture ELISA. A similar event was reported in the case of development of Crimean–Congo hemorrhagic fever virus (CCHFV) N protein detection ELISA system. The presence of antibodies to CCHFV N protein in the samples inhibited the reactivity of MAb with antigens in the CCHFV N protein Ag-capture ELISA [24]. Direct evidence of an inhibitory effect on Ag detection by anti-N Abs has been provided by experiments using mixtures of viremic serum with increasing amounts of immune serum [44]. Our data indicate significantly high IgG levels in the serum samples of Ag-capture ELISA negative patients. Based on these findings, the underlying immune status of patients may be characterized using this assay. It is concluded that the Ag-capture ELISA developed is available for serum samples collected during the early phase of SFTS before antibody responses become detectable.

Furthermore, the specific reaction of the MAbs to SFTSV antigens in tissues of patients with SFTS was confirmed (Fig 1B). Therefore, the MAbs were demonstrated to be of use in the detection of SFTSV antigen in the autopsied materials for SFTS diagnosis with IHC.

In this study, novel MAbs to SFTSV N protein were generated. The Ag-capture ELISA used for the MAbs in detecting SFTSV in the serum samples of the SFTS suspected patients was developed. Furthermore, MAbs were applied for the detection of SFTSV antigen in autopsied materials. These SFTSV antigen detection methods were useful for SFTS diagnosis.

## Supporting Information

S1 Fig The Ag-capture ELISA using both MAbs (9D3 and 2D11) as capture antibody is less sensitive than that using MAb 9D3 alone. The Ag-capture ELISA were performed by using MAb 9D3 or a combination of MAbs 9D3 and 2D11 as capture antibodies. Sera from rabbit immunized with SFTSV rN was used as a detecting antibody. The detection limits of authentic SFTSV (YG1 strain) by the Ag-capture ELISA were shown.
(PDF)

S2 Fig MAbs 9D3 and 2D11 do not react to Heartland virus N protein. The cDNA encoding Heartland virus (HRTV)-N protein (nucleotide position 1006–1743 of segment S, GenBank accession No. JX005842) amplified from HRTV RNA, kindly gifted from Dr. H. Ebihara (National Institute of Allergy and Infectious Diseases, Rocky Mountain Laboratories, Hamilton, Montana), was cloned into pCAG mammalian expression vector. The 293T cells transfected with the HRTV-N expression plasmid were used for IFA antigens. The antigens were incubated with MAb 9D3 or 2D11 at the concentration of 100ng/μl. Sera from rabbit immunized with SFTSV rN protein was used as a positive control. Anti-mouse or anti-rabbit IgG labeled with Alexa Fluor 488 (Life Technologies) was used for the 2^nd^ antibody.
(PDF)
