# Supplementary material for: Severe Fever with Thrombocytopenia Syndrome Virus Antigen Detection Using Monoclonal Antibodies to the Nucleocapsid Protein
Source: PLoS Negl Trop Dis. 2016 Apr 5;10(4):e0004595. doi: 10.1371/journal.pntd.0004595 (PMC4821557; doi:10.1371/journal.pntd.0004595)
Supplement: S1 Fig — The Ag-capture ELISA were performed by using MAb 9D3 or a combination of MAbs 9D3 and 2D11 as capture antibodies. Sera from rabbit immunized with SFTSV rN was used as a detecting antibody. The detection limits of authentic SFTSV (YG1 strain) by the Ag-capture ELISA were shown. (PDF) [file pntd.0004595.s001.pdf]

S1 Figure

MAb	Reactivity of MAb with SFTSV YG1 ($TCID_{50}$/100μl)							
	15653	7827	3913	1957	978	489	245	122
9D3	+	+	+	+	-	-	-	-
9D3+2D11	+	+	-	-	-	-	-	-
