# Supplementary figures and images for: Severe Fever with Thrombocytopenia Syndrome Virus Antigen Detection Using Monoclonal Antibodies to the Nucleocapsid Protein
Source: PLoS Negl Trop Dis. 2016 Apr 5;10(4):e0004595. doi: 10.1371/journal.pntd.0004595 (PMC4821557; doi:10.1371/journal.pntd.0004595)

S2 Figure

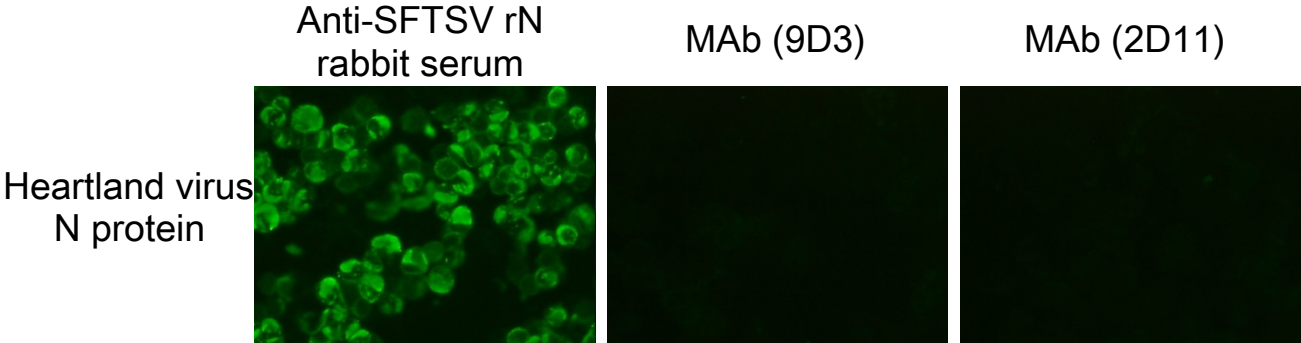

Supplement: S2 Fig — The cDNA encoding Heartland virus (HRTV)-N protein (nucleotide position 1006–1743 of segment S, GenBank accession No. JX005842) amplified from HRTV RNA, kindly gifted from Dr. H. Ebihara (National Institute of Allergy and Infectious Diseases, Rocky Mountain Laboratories, Hamilton, Montana), was cloned into pCAG mammalian expression vector. The 293T cells transfected with the HRTV-N expression plasmid were used for IFA antigens. The antigens were incubated with MAb 9D3 or 2D11 at the concentration of 100ng/μl. Sera from rabbit immunized with SFTSV rN protein was used as a positive control. Anti-mouse or anti-rabbit IgG labeled with Alexa Fluor 488 (Life Technologies) was used for the 2nd antibody. (PDF) [file pntd.0004595.s002.pdf]
